# Zoledronic Acid Modulation of TRPV1 Channel Currents in Osteoblast Cell Line and Native Rat and Mouse Bone Marrow-Derived Osteoblasts: Cell Proliferation and Mineralization Effect

**DOI:** 10.3390/cancers11020206

**Published:** 2019-02-11

**Authors:** Rosa Scala, Fatima Maqoud, Mariacristina Angelelli, Ramon Latorre, Maria Grazia Perrone, Antonio Scilimati, Domenico Tricarico

**Affiliations:** 1 Section of Pharmacology, Department of Pharmacy-Pharmaceutical Sciences, University of Bari, Via Orabona 4, I-70125 Bari, Italy; rosa.scala@uniba.it (R.S.); fatima.maqoud@uniba.it (F.M.); m.angelelli@hotmail.it (M.A.); 2 Facultad de Ciencias, Centro Interdisciplinario de Neurociencia de Valparaíso, Universidad de Valparaíso, Valparaíso 2366103, Chile; ramon.latorre@uv.cl; 3 Medicinal Chemistry Section, Department of Pharmacy-Pharmaceutical Sciences, University of Bari, Via Orabona 4, I-70125 Bari, Italy; mariagrazia.perrone@uniba.it

**Keywords:** TRPV1 channel, zoledronic acid, voltage dependent potassium channel, osteoblast, osteoclast, bisphosphonates, cell proliferation, mineralization

## Abstract

Bisphosphonates (BPs) reduce bone pain and fractures by balancing the osteoblast/osteoclast ratio. The behavior of ion channels in the presence of BPs is not known. To investigate this, the effect of zoledronic acid BP (ZOL) (3 × 10^−8^ to 5 × 10^−4^ M) treatment, on ion channels, cell proliferation, and mineralization, has been investigated on preosteoclast-like cells, RAW264.7, preosteoblast-like cells MC3T3-E1, and rat/mouse native bone marrow-derived osteoblasts. In whole-cell patch clamp on cell line- and bone marrow-derived osteoblasts, ZOL potentiated outward currents. On RAW264.7, ZOL (10^−4^ M)-evoked current was reduced by the Kv channel blocker tetraethylammonium hydrochloride (TEA), but not by the selective TRPV1-channel antagonist capsazepine. On MC3T3-E1 cells and bone marrow-derived osteoblasts, ZOL-evoked current (5 × 10^−8^ to 10^−4^ M) was reduced by capsazepine, whereas the selective TRPV1-channel agonist capsaicin potentiated the control current. In the cell proliferation assay, 72 h incubation of RAW264.7 and MC3T3-E1 cells with ZOL reduced proliferation, with IC_50_ values of 2.62 × 10^−7^ M and 2.02 × 10^−5^ M, respectively. Mineralization of MC3T3-E1 cells and bone marrow-derived osteoblasts was observed in the presence of capsaicin and ZOL (5 × 10^−8^–10^−7^ M); ZOL effects were antagonized by capsazepine. In summary, the ZOL-induced activation of TRPV1 channel mediates the mineralization of osteoblasts and counterbalances the antiproliferative effects, increasing the IC_50_. This mechanism is not operative in osteoclasts lacking the TRPV1 channel.

## 1. Introduction

Bone tissue is one of the preferred sites for metastasis in patients with advanced malignancy, especially in case of prostate, breast cancer, and multiple myeloma. Tumor cells prefer to migrate into heavily vascularized areas of the skeleton, such as the red marrow of the long bones, sternum, pelvis, ribs, and vertebrae [1], because of the support of their microenvironment. The bone microenvironment is considered a fertile “soil”, containing cytokines and growth factors that stimulate the growth of the cancer cells and whose structure can be a protective environment for dormant tumor cells [2]. The presence of cancer metastasis to bone is responsible for an uncoupled bone remodeling, leading to an imbalance between osteoclast-driven bone resorption and osteoblast-driven bone deposition [2] and, thereby, causing impaired mobility, pathologic massive fractures, spinal cord compression, bone marrow aplasia, hypercalcemia, and especially severe inflammatory and mechanical bone pain that significantly reduce the patient’s quality of life [3].

It is noteworthy that once cancer has spread to bone tissue, it can rarely be cured. The current standard of care for patients with bone loss due to osteolytic bone metastases includes antiresorptive therapy that can shrink or slow the growth of bone metastases and contribute to reducing bone pain and the risk of pathologic fractures, but that is not curative [2]. Among the clinically available drugs, bisphosphonates (BPs) are the most used bone-specific antiresorptive agents, and represent the first-line choice to treat osteolytic bone metastases as well as skeletal pathological conditions characterized by an alteration of bone density and thickness [4]. Since BPs can avidly bind to exposed bone mineral, their highest concentration is likely to be in the osteoclast-mediated resorption lacunae. Consequently, BPs are internalized by osteoclasts, where they act as potent inhibitors of farnesyl pyrophosphate synthase, a key enzyme of the mevalonate pathway, involved in bone resorption, affecting osteoclast morphology and finally inducing their apoptosis [5,6].

Besides the beneficial effects deriving from the interaction of the BPs with osteoclasts, these drugs also show antimyeloma and antitumor activity, leading to the increased overall survival of patients with various malignancies [7]. Indeed, BPs are responsible for a direct effect on cancer cells, interrupting the vicious cycle of increased osteolysis coupled with increased tumor growth, thus preserving bone health and delaying bone lesion progression. For example, third-generation bisphosphonates (i.e., zoledronic acid) can inhibit growth, migration, and matrix-associated invasion of breast cancer cells [3]. In vitro, human prostate cancer PC3 cells and human osteosarcoma MG63 cells treated with zoledronic acid, alendronate, or risedronate [6] show attenuated proliferation.

Although the effects of BPs for therapeutic intervention in bone metastases appear to be linked to the modulation of osteoclast activity and reduction of tumor cell proliferation, in the last years there has been increasing interest in BPs’ effects on osteoblasts and osteocytes, which led to uncovering their capability to promote increased mineralization, thereby improving bone density and thickness. In fact, BPs can prolong osteoblast lifespan [8], inducing pro-survival and pro-differentiation effects between concentrations of 10^−9^ M to 10^−6^ M [9], lower than the pro-apoptotic agents (over 10^−5^ M) [10]. However, the mechanisms of action which underpin these effects in osteoblasts are under debate, and different hypotheses have been tested. BPs appeared to be responsible for decreasing receptor activation of NF-κB ligand (RANKL) expression, and increasing RANKL decoy receptor osteoprotegerin (OPG) expression, causing an alteration in pro-differentiation, numbers, activity, and survival of osteoclasts [11]. Some experimental studies suggested that BPs can prevent osteoblast and osteocyte apoptosis, in vitro and in vivo, through the modulation of connexin 43 (Cx43), a member of the connexin family of proteins expressed in both osteoblasts and osteocytes [12]. More recently, it was also shown that protein tyrosine phosphatases (PTPs) can be BP targets in osteoblasts, and that this interaction leads to osteoblast proliferation, modulation of cytosolic Ca^2+^ levels in osteoblasts, as well as their maturation and differentiation [13]. Despite these advances, there are still interesting mechanistic issues to be solved. The involvement of new cell surface targets to explain how bisphosphonates block apoptosis of osteocytes, osteoblasts, and chondrocytes inducing cell proliferation at very low concentrations cannot be excluded.

It is now evident that a wide variety of factors are involved in the balance between bone resorption and bone production. In full agreement with this, a large variety of ion channels are known to play a leading role in regulating bone homeostasis, including K^+^ channels and transient receptor potential (TRP) channels.

Mesenchymal stem cells, the precursors of osteoblasts, are known to express different potassium channels which include KATP channels, inward rectifier K^+^ (Kir) channels, and small-, intermediate-, and large-conductance Ca^2+^-activated K^+^ channels [14]. Ca^2+^-activated K^+^ channels contribute to the regulation of cell volume, resting membrane potential, and intracellular levels of Ca^2+^ ions, essential for the mineralization process [15]. Moreover, BK channels together with KATP channels are recognized to contribute to osteocalcin secretion while, in osteocytes, K channels are involved in paracrine signals and mechanotransduction [16]. For example, K_Ca_3.1 calcium-dependent potassium channels cause membrane hyperpolarization required to optimize membrane potential during Ca^2+^ entry, maintaining the driving force for Ca^2+^ entry that activates stem cell proliferation [17]. Instead, loss of function of the inwardly rectifying K^+^ channel Kir2.1 impairs both osteoblastic and chondrogenic processes, leading to the rare Andersen’s syndrome [18]. Furthermore, we previously demonstrated that large-conductance Ca^2+^-activated K^+^ (BK) channels are involved in cell proliferation, since the incubation of SH-SY5Y cells with BK channel antagonists paxilline, iberiotoxin, and resveratrol was associated with a reduction of cell proliferation, reducing cell diameter and inducing AKT1p^ser473^ dephosphorylation [19]. 

Osteoblasts were also shown to express different kind of TRP channels, a very large family of cation-non-selective ion channels, differentially permeable to Ca^2+^ and Mg^2+^. Some specific TRP channels, including TRPM7, TRPV1, TRPV5, and TRPV6, appear to be involved in cellular Ca^2+^ and Mg^2+^ ion influx, thus regulating proliferation, differentiation, secretion, and apoptosis processes [20]. TRPV1, TRPV2, TRPV4, and TRPV5 are shown to be involved in osteoclastogenesis, TRPV4 is involved in chondrocyte differentiation and functioning, whereas TRPV1 appears to have a role in osteoblastogenesis and bone pain sensation [21]. Furthermore, it was recently shown that the activation of TRPV1 using capsaicin enhances osteoclast formation in bone marrow cultures stimulated with receptor activator of NFκB ligand (RANKL) [22]; TRPV1 antagonists can inhibit osteoclast formation in vitro, whereas on osteoblasts, the inhibition of TRPV1 is linked to the decreasing of alkaline phosphatase activity and bone nodule formation [23]. It was observed that blockers of TRPV1 might also reduce the pain associated with inflammatory arthritis and bone metastasis [23]. More recently, it was found that low-level laser irradiation (LLLI) induces bone formation and extracellular calcification of osteoblasts by upregulating proliferation and differentiation via TRPV1, and that LLLI-induced osteoblast proliferation can be inhibited by capsazepine [24]. Furthermore, TRPV5 knockout mice are found to be skeletally undeveloped and when treated with alendronate, a second-generation bisphosphonate, they experienced upregulation of TRPV5 in bone together with the decreased resorptive capacity of TRPV5(−/−) osteoclasts in vitro [25], thereby suggesting that TRPV5 also has an important role in osteoclast function affecting the mineralization process [26]. TRPM7 gene expression is shown to be increased after the osteoblastic differentiation of MC3T3-E1 murine preosteoblast-like cells, suggesting a key role for this channel in Ca^2+^/Mg^2+^ ions homeostasis [27]. 

Despite these considerations, BP effects on ion channels and the possible correlation between current modulation and BPs’ therapeutic effects on bone cell survival and osteoblast activity—essential for preserving bone homeostasis also in the presence of metastases, maintaining bone density and thickness and reducing bone pain sensation—have never been reported. The effects of zoledronic acid (ZOL) (3 × 10^−8^ to 5 × 10^−4^ M) on ion channels, especially potassium and TRP ion channels, cell proliferation, and mineralization were investigated on RAW264.7 preosteoclast-like cells, MC3T3-E1 preosteoblast-like cells, and rat/mouse native bone marrow-derived osteoblasts, and the obtained results are described below.

## 2. Results

### 2.1. In Vitro Cell Viability Experiments on RAW264.7 and MC3T3-E1 Cell Lines

We first compared the effects of three clinically used bisphosphonates (alendronate (ALE), risedronate (RIS), and zoledronic acid (ZOL)) on preosteoclast-like cells RAW264.7 and preosteoblast like cells MC3T3-E1 at different concentrations (3 × 10^−8^ to 5 × 10^−4^ M), to evaluate their capability in inducing antiproliferative effects on osteoclasts and pro-survival effects on osteoblasts.

**Figure 1:**
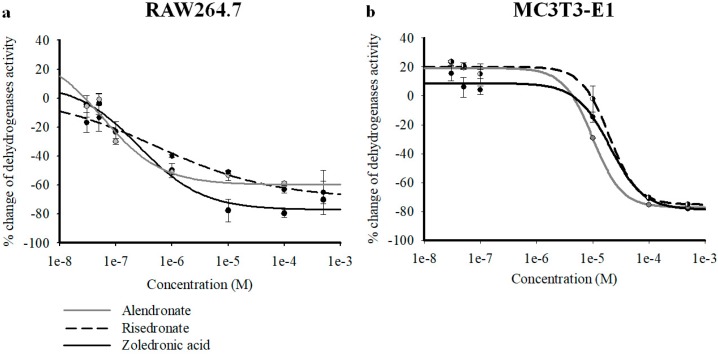
Percentage changes of dehydrogenase activity vs. alendronate (ALE), risedronate (RIS), and zoledronic acid (ZOL) concentrations in murine preosteoclast-like cells RAW264.7, and in murine preosteoblast-like cells MC3T3-E1. Cell dehydrogenase activity was measured using a colorimetric assay (Cell Counting Kit-8) after the incubation of the cells throughout 72 h. Each experimental point represents the mean ± SEM of at least three replicates. Data were fitted using the Hill equation (SigmaPlot 10). All three compounds were capable of causing a significant concentration-dependent reduction of cell dehydrogenase activity, with different efficacy and potency in (**a**) RAW264.7 cells and (**b**) MC3T3-E1 cells. The ZOL and ALE concentration–response relationships were shifted to the left on the log concentration axis in RAW264.7 cells. ZOL was more effective than ALE and RIS in reducing cell proliferation in RAW264.7 cells. All bisphosphonates (BPs) were capable of increasing cell dehydrogenase activity on MC3T3-E1 in the nanomolar concentration range.

On RAW264.7 cells, the three BPs strongly reduced intracellular dehydrogenase activity in the micromolar concentration range, as evaluated with CCK8 assays after 72 h of incubation. Concentration–response relationship analysis revealed a comparable capability of ZOL, RIS, and ALE in reducing dehydrogenase activity in CCK8 assay, as evaluated using one-way ANOVA analysis between drugs (*F* = 1.123). The maximal efficacy against RAW264.7 was, however, in favor of ZOL vs. the other BPs, with ZOL being more effective in inhibiting cell proliferation than ALE, as evaluated by Student *t*-test comparing the Emax percentage (%) of the different drugs (*p* < 0.05) (Table 1). Also, in preosteoblast-like cells MC3T3-E1, the three compounds were equally capable of reducing intracellular dehydrogenase activity in the micromolar concentration range, as evaluated using one-way ANOVA analysis between drugs (*F* = 1.111). The Hill coefficient was <1 for all the compounds in RAW264.7, whereas a slope >1 was calculated for MC3T3-E1. In MC3T3-E1 cells, all BPs caused a mild but not significant increase of dehydrogenase activity in the nanomolar concentration range (3 × 10^−8^ to 10^−7^ M) (Figure 1a,b).

**Figure 2:**
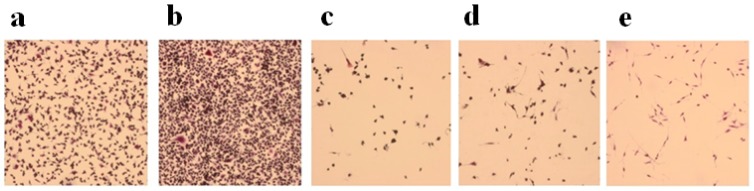
Representative images of polynucleated osteoclasts following RANK ligand treatment marked with TRAP, demonstrating the presence of tartrate-resistant acid phosphatase in osteoclasts. Inhibitory effects of osteoclastogenesis by alendronate (ALE), risedronate (RIS), and zoledronic acid (ZOL) tested at 10^−4^ M concentrations are shown at 10× magnification. Cells were treated with (**a**) normal medium; and medium with (**b**) RANKL, (**c**) ALE, (**d**) RIS, and (**e**) ZOL (Magnification is 10×).

In the osteoclastogenesis assay performed on differentiated RAW264.7 cells, ALE, RIS and ZOL were capable of inhibiting osteoclastogenesis in the micromolar concentration range after 7 days of incubation of the cells with RANKL. Randomly selected areas at each well were used to show the number of differentiated osteoclasts. At 10^−4^ M, the compounds were all capable of reducing the numbers of differentiated osteoclasts (Figure 2).

**Figure 3:**
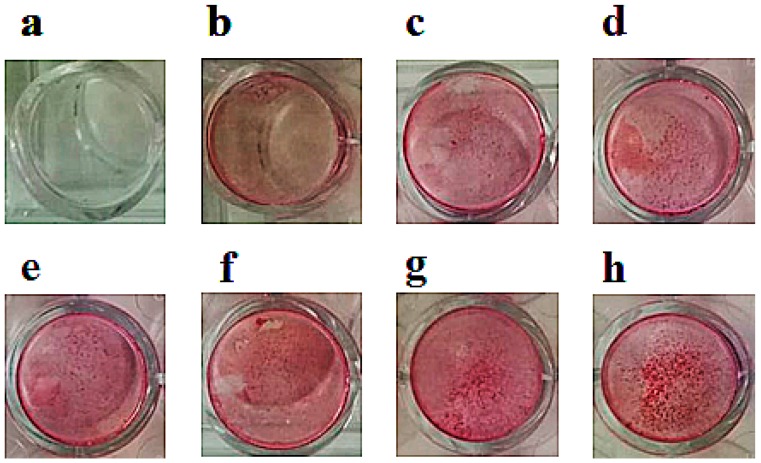
Mineralization assay with alizarin red S staining for calcium nodules after 15 days of incubation on MC3T3-E1 cells after treatments with alendronate (ALE), risedronate (RIS), and zoledronic acid (ZOL). Cells were treated with (**a**) normal medium, (**b**) mineralized medium, mineralized medium in the presence of (**c**) 3 × 10^−8^ M ALE, +38.68% ± 2.18% vs. mineralized medium in b, (**d**) 5 × 10^−8^ M ALE, +58.78% ± 6.08% vs. mineralized medium in b, (**e**) 3 × 10^−8^ M RIS, +45.13% ± 4.12% vs. mineralized medium in b, (**f**) 5 × 10^−8^ M RIS, +65.63% ± 5.22% vs. mineralized medium in b, (**g**) 3 × 10^−8^ M ZOL, +99.18% ± 31.28% vs. mineralized medium in b, (**h**) 5 × 10^−8^ M ZOL, +136.08% ± 21.48% vs. mineralized medium in **b**.

Then, BP effects in the nanomolar concentration range were investigated using the mineralization-related osteoblastogenesis assay, to evaluate the capability of drugs to modify calcium phosphate nodule formation. On differentiated MC3T3-E1 cell line, ZOL, RIS, and ALE were capable of inducing significant osteoblastogenesis and mineralization in the nanomolar concentration range (3 × 10^−8^ to 5 × 10^−8^ M). In our previous work, these concentrations were effective in inducing mineralization in cell lines [6]. At 5 × 10^−8^ M, ZOL was the most effective drug in inducing mineralization, which increased by +136.08% ± 21.48% compared to controls (number of replicates = 5) as determined by Student *t*-test (*p* < 0.05). At this concentration, RIS and ALE were less effective than ZOL in inducing nodule formation, causing an increase of +65.63% ± 5.22% and +58.78% ± 6.08% vs. controls group (*p* < 0.05) (number of replicates = 3), respectively. Nodule formation of calcium phosphate precipitate was visible after 10–15 days of incubation of cells with drugs in the mineralized medium (Figure 3). Instead, no effect of these drugs was observed in the micromolar concentration (data not shown).

Based on these results, ZOL appeared to be the most effective compound in modulating cell activity both in osteoblast and osteoclast cell lines. In fact, the calculated low IC_50_ MC3T3-E1/IC_50_ RAW264.7 ratio of ZOL of 77, and the osteoclastogenesis assay, revealed a strong selectivity of ZOL for osteoclasts with regard to the reduction of proliferation and the differentiation process. More remarkably, ZOL was able not only to increase dehydrogenase activity in preosteoblast-like cells MC3T3-E1 but, also, to induce notable mineralization in the nanomolar concentration range. Therefore, since ZOL appeared to be the most effective drug in all the performed assays, ZOL actions were further investigated on ion channel currents in whole-cell patch clamp on the target cells.

### 2.2. Characterization of Cation Channel Currents of RAW264.7 and MC3T3-E1 Cell Lines in Controls and in the Presence of ZOL

**Figure 4:**
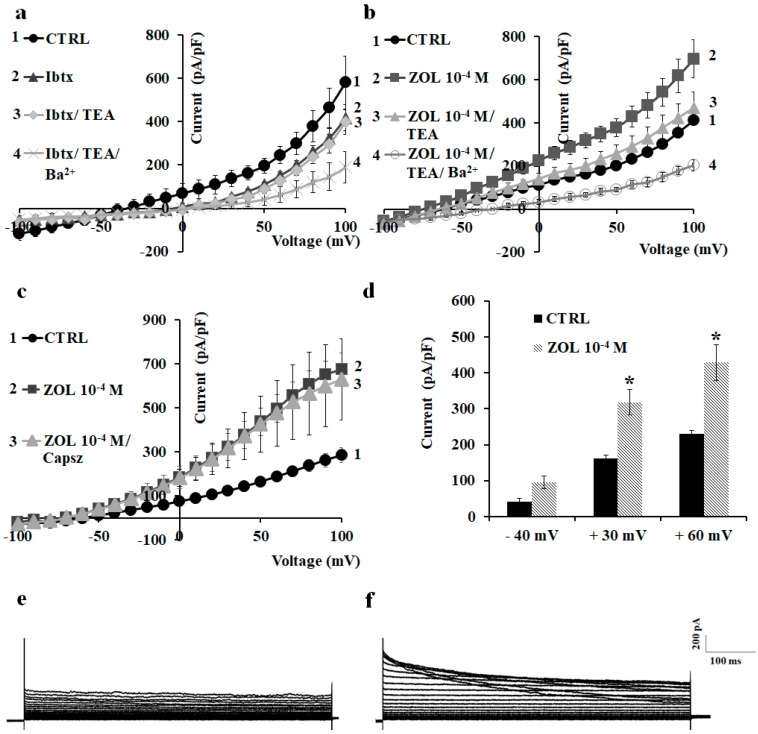
Characterization of the inwardly and outwardly macroscopic K^+^ currents recorded in RAW264.7 preosteoclast-like cells using a whole-cell configuration. Currents were recorded under physiological K^+^ ion concentration in the bath and pipette, and were obtained in response to voltage pulses from −100 to +100 mV in 10 mV steps, starting from HP = −60 mV (Vm). Cells of about the same size were selected for patch clamp experiments. Each point represented the mean ± SEM (*N* patches = 5–8). (**a**) Current–voltage relationships in response to the application of Ibtx (4 × 10^−7^ M); Ibtx and TEA (5 × 10^−3^ M); and Ibtx, TEA, and Ba^2+^ (5 × 10^−3^ M). (**b**) Macroscopic currents induced by zoledronic acid (ZOL) (10^−4^ M) in RAW264.7 inhibited by TEA (5 × 10^−3^ M). (**c**) Current response to capsazepine (Capsz) (10^−6^ M). This compound failed to reduce ZOL-evoked current. (**d**) Change of current amplitudes at −40, + 30, and + 60 mV (Vm) after ZOL application (10^−4^ M) on RAW264.7 cells. * Data significantly different with respect to the controls (*p* < 0.05; Student *t*-test). (**e**) Sample traces of control (CTRL) current and (**f**) ZOL (10^−4^ M)-evoked instantaneous current.

We first characterized the macroscopic currents on preosteoclast-like cells RAW264.7 in whole-cell patches. Using physiological K^+^ concentrations in the bath and pipette solutions, a hyperbolic current–voltage relationship can be recorded in RAW264.7 cells. The resting potential (Vm) of these cells was −44.49 ± 3.40 mV (number of cells = 48). The application of iberiotoxin (Ibtx), a selective BK channel blocker, to a final concentration of 4 × 10^−7^ M, reduced the cationic currents inducing cell depolarization. The co-incubation of the Ibtx with 5 × 10^−3^ M TEA did not induce a further reduction of the cationic currents, suggesting that BK channels carried at least 27.9% of the control currents. The co-incubation of the cells with Ibtx, TEA, and 5 × 10^−3^ M Ba^2+^ caused an almost complete reduction of the control currents, suggesting the contribution of inward-rectifying potassium (Kir) channels and outward-rectifying K^+^ channels to the control currents (Figure 4a).

The application of 10^−4^ M ZOL on the RAW264.7 cells induced a sustained enhancement of the cationic currents both at positive and negative membrane potentials. The co-incubation of the ZOL solution with 5 × 10^−3^ M TEA caused a partial reduction of the ZOL-activated currents, suggesting that Kv/BK channels could carry the ZOL-activated currents. The co-incubation of the cells with ZOL, TEA. and 5 × 10^−3^ M Ba^2+^ caused a further reduction of the currents below controls (Figure 4b). The application of 10^−6^ M capsazepine, a selective TRPV1 channel antagonist, failed to reduce ZOL-evoked currents (Figure 4c).

Under this experimental condition, 10^−4^ M ZOL enhanced current amplitude both at negative and positive voltages (Figure 4d). In detail, 10^−4^ M ZOL enhanced currents amplitude by +14.93% at −40 mV, +63.05% at +30 mV, and +110.29% at +60 mV (Vm). No effect of ZOL was observed on the current amplitude at nanomolar concentrations.

In agreement with our functional observation, the mRNA levels and protein of TRPV1 gene was not detected by others in this cell line [28,29,30].

**Figure 5:**
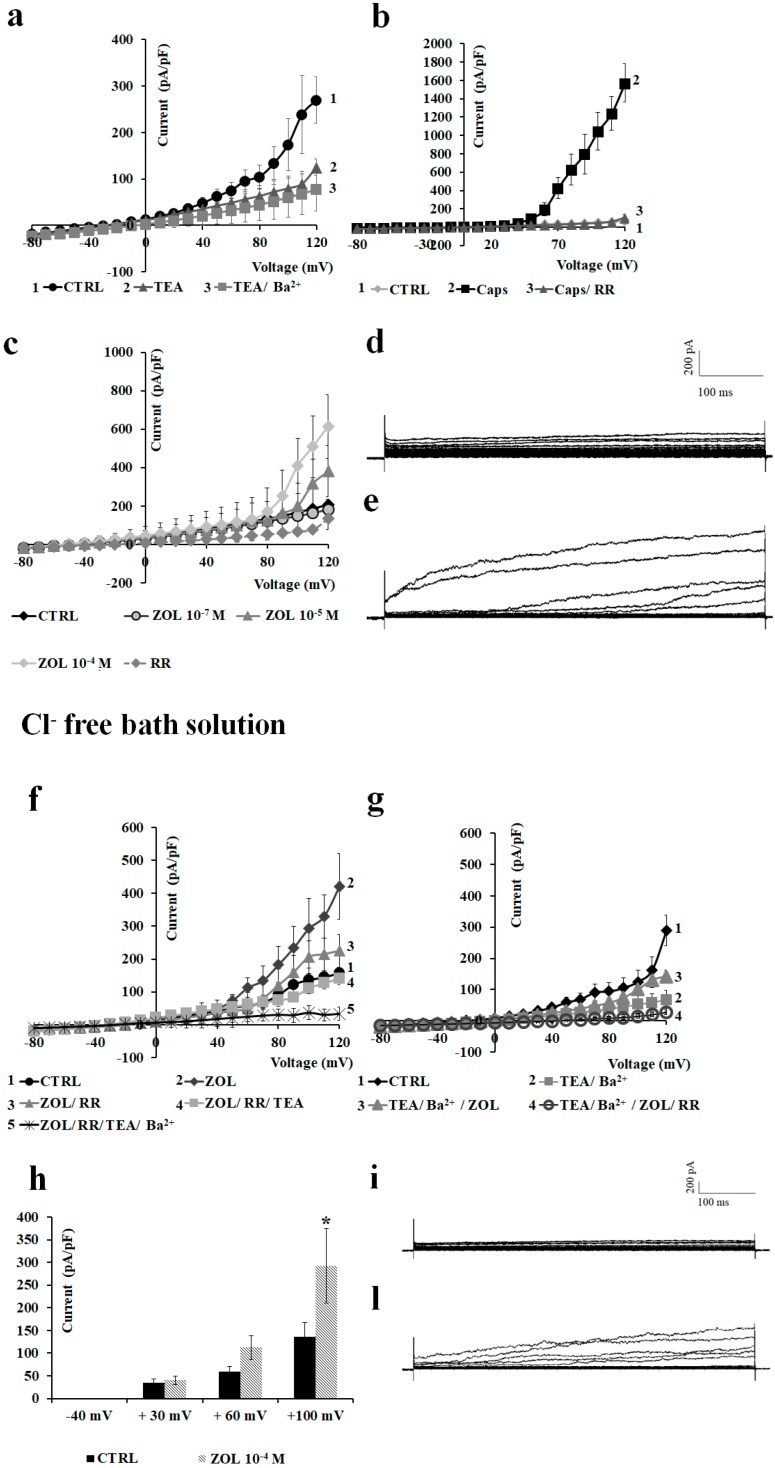
Electrophysiological characterization of macroscopic K^+^-currents and transient receptor potential (TRP) channel currents recorded in preosteoblast-like cells MC3T3-E1 in whole-cell configuration. The current was recorded using physiological K^+^ concentration in the bath and pipette, and was obtained in response to voltage pulses from −80 to +120 mV in 10 mV steps, starting from HP = −60 mV. Each point represented the mean ± SEM of 5–7 patches. (**a**) Inhibitory response of control currents to TEA (5 × 10^−3^ M) and Ba^2+^ (5 × 10^−3^ M) that partially reduced the currents. (**b**) Activating response of control currents to capsaicin (Caps) (10^−5^ M) that was fully inhibited by ruthenium red (RR) (10^−5^ M). (**c**) Activating response of control currents to ZOL (10^−5^ M and 10^−4^ M) concentrations that was fully inhibited by RR at positive voltage membrane. (**d**) Sample traces of CTRL current and (**e**) ZOL (10^−4^ M)-evoked instantaneous current. (**f**) Activating response of control currents to ZOL (10^−4^ M) in Cl^−^-free bath solution that was antagonized by RR (10^−5^ M), RR and TEA (5 × 10^−3^ M), and RR/TEA and Ba^2+^ (5 × 10^−3^ M). (**g**) ZOL induced activation of the currents obtained following incubation of the cells with the unselective K^+^-channel blockers Ba^2+^ (5 × 10^−3^ M) and TEA (5 × 10^−3^ M) in the Cl^−^-free bath solution. (**h**) Change of current amplitudes at −40, +30, + 60, and +100 mV (Vm) after ZOL application (10^−4^ M) on MC3T3-E1 cells in the Cl^−^-free bath solution. * Data significantly different with respect to the controls at *p* < 0.05 as determined by Student *t*-test. (**i**) Sample traces of CTRL current and (**l**) ZOL (10^−4^ M)-evoked instantaneous current in Cl^−^-free bath solution.

Next, we characterized the macroscopic currents recorded in the preosteoblast-like cells MC3T3-E1 in whole-cell patches in the same experimental conditions. The Vm of these cells in the control condition was −29.45 ± 2.37 mV (number of cells = 45). At −80 mV (Vm) the current amplitude was −18.2 ± 0.49 pA (number of cells = 45), while a large outward current of +105.1 ± 24.9 pA (number of cells = 45) was recorded at positive membrane potentials. TEA at 5 × 10^−3^ M reduced the current amplitude, and co-incubation with 5 × 10^−3^ M Ba^2+^ caused an incomplete reduction of voltage-dependent currents, suggesting the presence of other channels, beside BK and Kv channels, contributing to the control current (Figure 5a). The application of 10^−5^ M capsaicin, a well-known transient receptor potential vanilloid 1 (TRPV1) channel agonist, elicited large outward currents. Ruthenium red (RR), a non-selective TRP channel blocker, completely reduced the capsaicin-induced currents at 10^−5^ M, supporting the idea that the TRPV1 channel is functionally active in these cells (Figure 5b).

The application of ZOL on the macroscopic currents of MC3T3-E1 cells induced a concentration-dependent potentiation of these currents. Both 10^−5^ M and 10^−4^ M ZOL increase outward currents. ZOL-evoked currents were almost completely abolished after the application of 10^−5^ M ruthenium red (Figure 5c). ZOL at 10^−4^ M enhanced current amplitude at positive voltages, with a stronger activation at membrane voltage >+90 mV (Vm) (Figure 5c). ZOL at 10^−4^ M mildly enhanced current amplitude by +9.35% at +30 mV, and +13.51% at +60 mV (Vm), and the inward current at −80 mV (Vm) by +12.1%.

In low Cl^−^ solution, 10^−4^ M ZOL applied to the cells confirmed its ability to potentiate outward currents, which could be partially reduced by 10^−5^ M ruthenium red by −55.39%, and were fully reduced after the application of TEA (5 × 10^−3^ M) and Ba^2+^ (5 × 10^−3^ M) (Figure 5f). Under these experimental conditions, 10^−4^ M ZOL was still capable of inducing a notable activation at membrane voltages >+80 mV (Vm). In particular, 10^−4^ M ZOL enhanced currents by +4.46% at +30 mV (Vm), significantly enhanced current amplitude by + 54.83% at +60 mV (*p* < 0.05) and +725.08% at +100 mV (Vm) vs. controls (*p* < 0.05) (Figure 5h).

The effects of ZOL were investigated on leak currents recorded following incubation of the cells with the unselective K^+^-channel blockers Ba^2+^ (5 × 10^−3^ M) and TEA (5 × 10^−3^ M) in the Cl^−^-free bath solution. We found that the application of ZOL (10^−4^ M), even in the presence of these blockers, potentiated the outward currents that were antagonized by ruthenium red (10^−5^ M), indicating that ZOL-activated currents were carried not only by K^+^ channels, but also by other different cationic channels, possibly TRP (Figure 5g).

**Figure 6:**
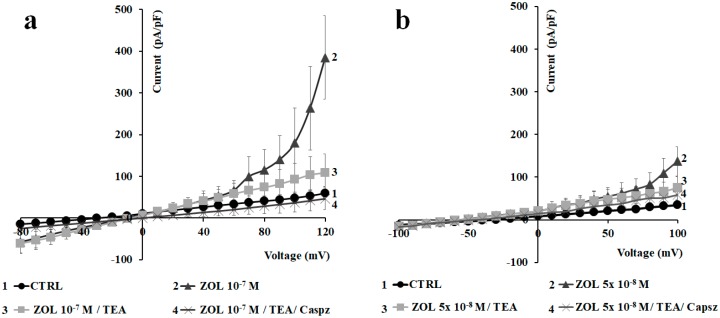
Effects of 5 × 10^−8^ to 10^−7^ M zoledronic acid after 10 and 30 min of incubation time on preosteoblast-like cells MC3T3-E1 using whole-cell patches. Current was recorded using physiological K^+^ concentration in the bath and pipette, starting from HP = −60 mV (Vm). Cells of about the same size were selected for patch clamp experiments. (**a**) Zoledronic acid (ZOL) (10^−7^ M) was able to induce a strong activation of outward currents in MC3T3-E1 cells in response to voltage pulses from −80 to +120 mV (Vm), in 10 mV steps, after about 30 min of incubation using whole-cell patches. ZOL-evoked current was reduced after the application of TEA (5 × 10^−3^ M) and fully closed after the application of capsazepine (10^−6^ M). Each point represented the mean ± SEM of 3–10 patches. (**b**) ZOL (5 × 10^−8^ M) was able to potentiate outward currents in MC3T3-E1 cells in response to voltage pulses from −100 to +100 mV (Vm), in 10 mV steps, after about 10 min of incubation using whole-cell patches. ZOL-evoked current was reduced after the application of TEA (5 × 10^−3^ M) and capsazepine (10^−6^ M). Each point represented the mean ± SEM of 4–10 patches.

Since ZOL, at 10^−7^ M concentration, did not produce notable activating effects on macroscopic currents following acute application of the drug solution, we also tested the effects of ZOL at this concentration as a function of time in the same MC3T3-E1 cells. After about 30 min of incubation time, ZOL (10^−7^ M) caused an enhancement of the outward currents (number of cells = 10). In the majority of the investigated cells, the ZOL-evoked current was markedly reduced by TEA (5 × 10^−3^ M) by −66.36% at +100 mV (Vm); a further and full reduction of the current was observed with capsazepine (10^−6^ M) (Figure 6a). Cells responsive to ZOL (10^−7^ M) were also responsive to capsaicin (10^−5^ M). Instead, no activation was observed in cells not responsive to capsaicin (data not shown) after a long-term incubation time with ZOL (10^−7^ M). These findings indicate that currents activated by ZOL (10^−7^ M) involved TRPV1 and/or Kv/BK channels.

Similar effects were observed after the application of ZOL (5 × 10^−8^ M) on the same MC3T3-E1 cells. After about 10 min of incubation time, ZOL (5 × 10^−8^ M) caused an enhancement of the outward currents (number of cells = 16). In the majority of the investigated cells, the ZOL-evoked current was markedly reduced by TEA (5 × 10^−3^ M) by −60.91% at +100 mV (Vm), with a further reduction of the current by −15.42% at +100 mV (Vm) after the application of capsazepine (10^−6^ M) (Figure 6b). In some other cells (number of cells = 8), 5 × 10^−8^ M ZOL failed to activate the current. These findings indicate that 5 × 10^−8^ M concentration could be the threshold ZOL concentration to potentiate the Kv/BK and TRPV1 channels in the MC3T3-E1 osteoblast cell line in whole-cell configuration. 

In agreement with this observation, the mRNA level of the TRPV1 gene was found to be elevated in this cell line and osteoblasts [31,32].

### 2.3. Pharmacological Characterization of the Whole-Cell Currents of Native Mesenchymal Stem Cells (MSCs) from Bone Marrow of Mouse and Rat

**Figure 7:**
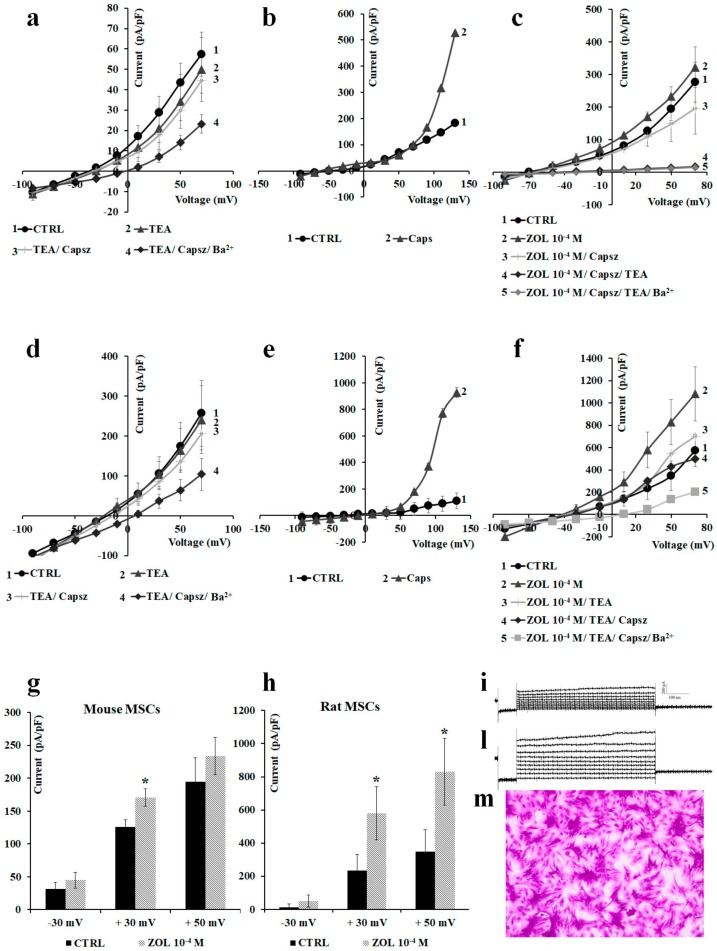
Pharmacological characterization of the inwardly and outwardly macroscopic K^+^ currents and transient receptor potential (TRP) channel currents recorded in mouse and rat mesenchymal stem cells (MSCs) in whole-cell configuration. Current was recorded using physiological K^+^ concentration in the bath and pipette, and was obtained in response to voltage pulses from −90 to + 70 mV in 20 mV steps, starting from HP = −60 mV (mV). Each point represented the mean ± SEM of 1–10 patches. (**a**) In mouse MSCs (number of patches = 1–5 patches) and (**d**) rat MSCs (number of patches = 1–5 patches), there was an inhibitory response of the control currents to TEA (5 × 10^−3^ M) and capsazepine (Capsz) (10^−6^ M). (**b**) In murine MSCs and (**e**) rat MSCs, there was an activating response of control currents to capsaicin (Caps) (10^−5^ M). (**c**) In murine MSCs and (**f**) rat MSCs, there was an activating response of control currents to ZOL (10^−4^ M) that was fully inhibited by co-application of capsazepine (10^−5^ M) and TEA (5 × 10^−3^ M). (**g**) Change of current amplitude at −30, +30, and +50 mV (Vm) after ZOL application (10^−4^ M) on mouse MSCs and (**h**) rat MSCs. * Data are significantly different with respect to the controls for *p* < 0.05 as determined by Student *t*-test. (**i**) Sample traces of control (CTRL) current and (**l**) ZOL-evoked instantaneous current in rat MSCs. (**m**) Image of murine MSCs as observed by Olympus CX41 4× magnification after crystal violet staining.

We next investigated whether ZOL was also able to activate currents in native mesenchymal stem cells (MSCs) isolated from both mouse and rat bone marrow [33]. Firstly, macroscopic currents were pharmacologically characterized in MSCs isolated from mouse bone marrow cells. Using asymmetrical, physiological K^+^ concentration in the bath and pipette solutions, a hyperbolic current was observed that crossed the voltage axis −40 ± 8.28 mV (number of cells = 15) and −36.08 ± 3.78 mV (Vm) (number of cells = 12), representing the resting potential of the mouse and rat cells, respectively. TEA (5 × 10^−3^ M) as well as capsazepine (10^−6^ M) reduced current amplitudes to control values suggesting the contribution of Kv/BK and TRPV1 channels to the total current in either cell population (Figure 7a–d). The application of capsaicin (10^−5^ M) potentiates the hyperbolic current, supporting the idea that TRPV1 channels are functionally active in these cells (Figure 7b–e). Both mouse MSCs and rat MSCs, therefore, showed similar properties regarding the response to channel modulators, even if mouse cells showed lower current amplitude in control conditions. 

On these cells, ZOL (10^−4^ M) elicited large, outward currents that were blocked by co-application of capsazepine (10^−6^ M) and TEA (5 × 10^−3^ M), further confirming ZOL’s capability of enhance TRPV1 and Kv/BK channel currents (Figure 7c–f). Especially on mouse MSCs, ZOL (10^−4^ M) enhanced current amplitude by +5.37% at −30 mV, +29.82% at +30 mV, and +47.69 at +50 mV vs. controls, and on rat MSCs by +6.94 at −30 mV, +101.37% at +30 mV, and +212.67% at +50 mV. On rat, TEA (5 × 10^−3^ M) caused a reduction of −75.35%, and capsazepine (10^−6^ M) completely closed ZOL-evoked currents, supporting the idea that Kv/BK channels are responsible for most of the ZOL-activated currents in these cells (Figure 7g,h). ZOL (10^−4^ M) also enhanced the inward current by +81.9% at −90 mV in mice and +54.3% in rat, and these effects were fully inhibited by TEA and capsazepine (Figure 7c–f).

### 2.4. Effects of Ion Channel Modulators on ZOL-Induced Mineralization on Osteoblasts

**Figure 8:**
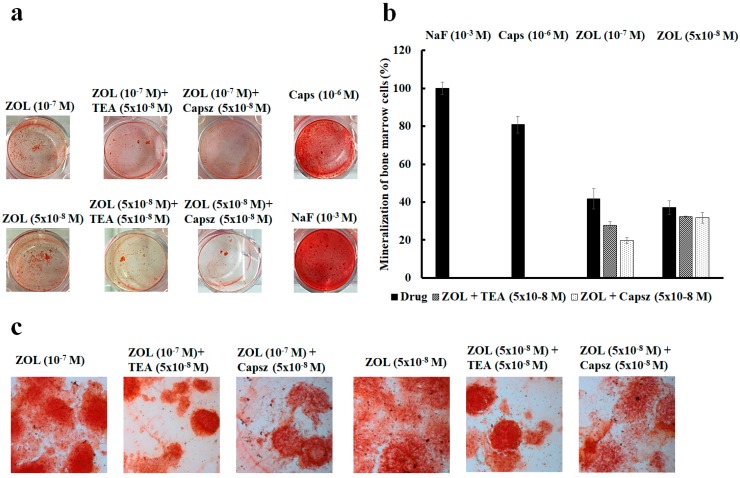
(**a**) Mineralization assay with alizarin red S staining for calcium nodules upon treatment with different ion channel modulators in the presence or absence of ZOL after 15 days of incubation in murine bone marrow cells. (**b**) ZOL-induced mineralization respect to NaF (10^−3^ M). ZOL capability to mineralize is reduced by co-application with capsazepine and TEA. (**c**) Alizarin red S staining for calcium nodules after 21 days of incubation in MC3T3-E1 cells after treatments with different ion channel modulators in the presence or absence of ZOL. Images were captured using an Olympus CX41 biological system microscope at 40× magnification.

The effects of ion channels modulators on the mineralization process induced by ZOL was evaluated using the mineralization assay both in mouse and rat bone marrow-derived osteoblasts (Figure 8a,b) and MC3T3-E1 cells (Figure 8c). The extracellular matrix Ca^2+^ deposits used for mineralized nodule formation were stained with alizarin red S dye, which combines with Ca^2+^ ions to stain calcified nodules bright red, after 15 days of culture in the mineralized medium in the presence of ZOL (5 × 10^−8^ to 10^−7^ M) with or without channel inhibitors.

On native bone marrow cells, the effects of TEA (5 × 10^−8^ to 5 × 10^−3^ M) and capsazepine (5 × 10^−8^ to 10^−7^ M) on ZOL-induced mineralization were investigated at different concentrations after 15 days of incubation of the culture in the mineralized medium. The co-incubation of TEA (5 × 10^−8^ M) with ZOL (5 × 10^−8^ to 10^−7^ M) reduced the capability of ZOL to induce mineralization in 4 out of 10 experiments on rat and mouse bone marrow-derived osteoblasts. TEA reduced the ZOL-induced mineralization by −29.64% ± 8.52% (number of experiments = 4). Also, the co-incubation of capsazepine (5 × 10^−8^ M) with ZOL (5 × 10^−8^ to 10^−7^ M) reduced the capability of ZOL to induce mineralization in 8 out of 10 experiments on these cells. Capsazepine antagonized the ZOL-induced mineralization by −29.50% ± 5.01% (number of experiments = 8).

Moreover, capsaicin (10^−6^ M) alone on murine bone marrow cells was responsible for inducing strong mineralization leading to an increase of +209.19% ± 45.33% (number of experiments = 3) compared to the control conditions, suggesting a key role for the TRPV1 channel in mineralization and the osteoblastogenesis process. NaF (10^−3^ M) a well-known chemical inducing mineralization agent that led to a mineralization increase of +215% ± 58.39% (number of experiments = 3) compared to the control condition on mouse bone marrow-derived osteoblast cells (Figure 8a). The fact that ZOL at 10^−7^ M and 10^−8^ M concentrations showed equivalent effects in inducing mineralization, and capsazepine is effective against ZOL (10^−7^ M), can be explained by the fact that the threshold concentration of ZOL for inducing TRPV1-mediated mineralization is 10^−7^ M. This is also the threshold concentration required to activate TRPV1 and Kv/BK channels in osteoblasts in our experiments. Maximal mineralization is observed at 10^−7^ M of ZOL and, under these conditions, capsazepine is fully effective. 

The capability of ZOL in inducing mineralization was further compared to NaF (10^−3^ M) on mouse bone marrow-derived osteoblast cells. We found that ZOL at 5 × 10^−8^ and 10^−7^ M concentrations was less effective than NaF (10^−3^ M) in inducing mineralization. In these experiments, capsazepine was more effective than TEA in antagonizing the ZOL-induced mineralization on mouse bone marrow-derived osteoblast cells (Figure 8b).

A similar capability of TEA and capsazepine in antagonizing ZOL-induced mineralization was observed in MC3T3-E1 cells (Figure 8c).

It is evident that TEA alone at high concentrations caused a reduction of cell proliferation, thereby affecting mineralization after 15 days of incubation time. TEA at 5 × 10^−8^ M did not significantly affect, per se, mineralization of bone marrow cells. Also, the incubation of the rat and mouse bone marrow cells with capsazepine (5 × 10^−8^ to 10^−7^ M) reduced the proliferation at high concentrations while, at 5 × 10^−8^ M, this compound did not significantly affect proliferation. 

### 2.5. ZOL Effects on Oocytes Transfected with TRPV1 Channel Clone

**Figure 9:**
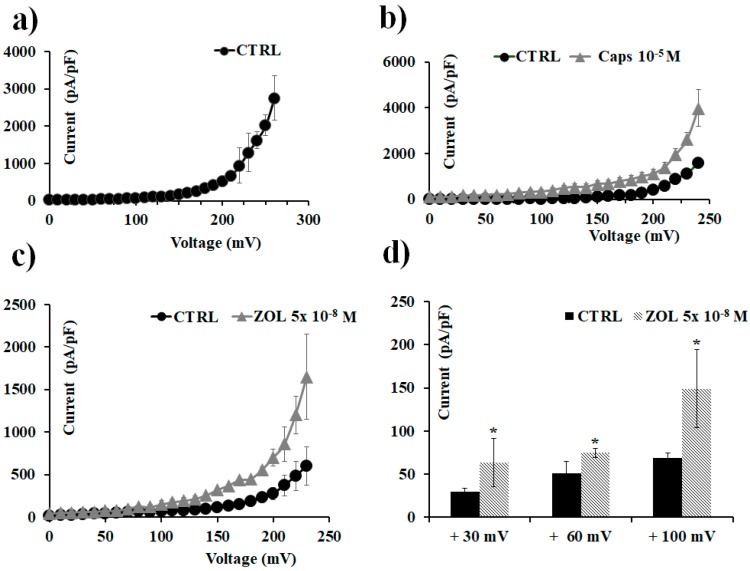
Transient receptor potential vanilloid 1 (TRPV1) channel currents recorded in *Xenopus laevis* oocytes in inside-out configuration, and capsaicin and ZOL effects. Current was recorded using symmetrical ion concentrations in the bath and pipette, and was obtained in response to a depolarization protocol, starting from HP = −60 mV (Vm). Each point represents the mean ± SEM. Temperature: 20 °C. (**a**) Control current was obtained in response to voltage pulses from 0 to 260 mV (Vm) in 10 mV steps. Each point is the average of measurements on 51 patches. (**b**) Macroscopic currents for TRPV1 induced by capsaicin (10^−5^ M) (number of patches = 7). (**c**) Macroscopic currents for TRPV1 induced by ZOL (5 × 10^−8^ M) in response to voltage pulses from 0 to + 230 mV (Vm) in 10 mV steps (number of patches = 3). (**d**) Change of currents amplitude at +30, +60, and +100 mV (Vm) after ZOL (5 × 10^−8^ M) application. * Data significantly different with respect to the controls for *p* < 0.05 as determined by Student *t*-test.

Finally, the effects of ZOL on TRPV1 channels were evaluated by using excised inside-out macropatches in *Xenopus* oocytes; it was found that ZOL was capable of activating TRPV1 currents when directly applied to the excised patch (Figure 9). In particular, ZOL (5 × 10^−8^ M) enhanced TRPV1 currents of +51.25% at +30 mV, +21.58% at +60 mV, and +32.06% at +100 mV (Vm) with respect to the current activated by capsaicin (10^−5^ M) at the same voltages. Therefore, ZOL at nanomolar concentration activates the TRPV1 channel current expressed in *Xenopus* oocytes, in excised macropatches, at negative and positive membrane voltages.

## 3. Discussion

In the present work, we show that zoledronic acid, at nanomolar concentrations, potentiates outward currents and induces cell proliferation and mineralization of MC3T3-E1 preosteoblast-like cells, and rat and mouse native bone marrow-derived osteoblasts. This drug at micromolar concentrations also has antiproliferative effects on RAW264.7 preosteoclast-like cells and MC3T3-E1 preosteoblast-like cells.

The zoledronic acid-induced mineralization, observed at nanomolar concentrations, is mediated by the activation of TRPV1 channel with the contribution of TEA-sensitive voltage-dependent K^+^ channels. These findings were supported by the fact that ZOL is capable of potentiating the whole-cell currents recorded in MC3T3-E1 cell and the mouse and rat bone marrow-derived osteoblasts, and these currents were partially antagonized by the selective TRPV1 blocker capsazepine and by the unselective Kv/BK channel blocker TEA. The ZOL activating action of the outward currents was observed in physiological conditions, as well as in Cl^−^-free bath solution, supporting the notion that the currents were carried by cations. Furthermore, the zoledronic acid-induced mineralization was prevented by capsazepine in 8 out of 10 experiments by −29.5%. TEA was also effective in 4 out of 10 experiments in antagonizing the mineralization induced by ZOL by −29.4%. These findings suggest that Kv/BK and TRPV1 channels contribute to the ZOL-induced mineralization by about 60%, while the residual 40% of mineralization is sustained by mechanisms involving, for instance, the activation of connexin 43 and the inhibition of intracellular protein tyrosine phosphatases [5,8,12,13]. 

Also, it was not possible to use specific BK channel blockers, such as Ibtx or paxilline, because of their irreversible blocking action of the BK channel associated with antiproliferative effects [19,34]. Also, it should be noted that other potent and selective TRPV1 antagonist are available; in our experiments, we use capsazepine to block TRPV1 channel because of the its well-known pharmacology and the lack of action on cell proliferation and mineralization. Despite that in our experiments we could not clearly identify the Kv channel type activated by ZOL, the best candidate is the BK channel. Functional coupling between TRPV1 and BK channels has indeed been reported in neurons, contributing to the modulation of pain [35].

The action of ZOL (5 × 10^−8^ M) on TRPV1 channel appears to be mediated by the direct interaction with the channel subunits, as demonstrated by the fact that this drug was capable of activating, in excised macropatches, the TRPV1 channel expressed in *Xenopus* oocytes. Currently, we do not know the location of the binding sites of ZOL on the channel subunits modulating channel function. It can be speculated that the binding site is located on the cytosolic face of the TRPV1 channel. This hypothesis is supported by the fact that ZOL, at nanomolar concentrations, is more effective in activating the TRPV1 current of a clone expressed in *Xenopus* oocyte in excised macropatches, rather than the TRPV1 whole-cell current recorded in the osteoblasts. Also, the activating effect of ZOL on the TRPV1 channel current at nanomolar concentrations is rescued following the long-term incubation of the osteoblasts with the drug.

At micromolar concentrations, ZOL and other BPs show antiproliferative effects associated with the inhibition of intracellular target/s, such as the human recombinant enzymes *h*FPPS and/or *h*GGPPS [6]. Here, we showed that, in preosteoblast-like cells MC3T3-E1, the BPs under investigation were equally capable of reducing intracellular dehydrogenase activity. The IC_50_ MC3T3-E1/IC_50_ RAW264.7 ratio used as a cell-selective indicator against RAW264.7 for RIS, ZOL, and ALE were 36.8, 77.0, and 169.0 in favor of RIS; while ZOL was the most effective drug in inducing the maximal reduction of cell proliferation on RAW264.7 vs. other BPs. The concentration–response curves of these drugs were relatively flat, as also demonstrated by the slope coefficient that was <1 for all the compounds in RAW264.7, suggesting a negative cooperativity between drugs at the binding site, whereas a slope >1 was calculated for MC3T3-E1, supporting the involvement of additional target-modulating BP effects on osteoblasts, such as TRPV1 channel. 

By contrast, on RAW264.7 cells, the ZOL-evoked currents were not responsive to capsazepine, suggesting that TRPV1 channels are not functionally active in these cells. Previous reports failed to show any evidence of TRPV1 channels in osteoclasts, both using immunoblotting analysis and RT-PCR [28,29,30], whereas RT-PCR analysis revealed that TRPV1 mRNA is expressed in MC3T3-E1 cells [31,32]. 

Moreover, in the nanomolar concentration range (3 × 10^−8^ to 10^−7^ M), the BPs failed to increase intracellular dehydrogenase activity on RAW264.7 cells in the cell proliferation assay. These findings suggest that the observed ZOL-activating action of TRPV1 channel is associated with cell proliferation and mineralization of osteoblasts with cytoprotection, explaining its selective action as an antiproliferative drug against the osteoclasts lacking TRPV1 channel current. 

We propose the following cascade of events: 

ZOL-induced transient activation of TRPV1 at nanomolar concentrations may lead to the influx of Ca^2+^ ions and osteoblast depolarization with activation of Kv and BK channels in the cells that maintain the membrane potentials with cytoprotection. This mechanism triggers the proliferation and mineralization of osteoblasts. It is expected that TRPV1, once activated by capsaicin and ZOL, would rapidly desensitize in response to persistent activation.

At micromolar concentrations, ZOL induces inhibition of intracellular *h*FPPS and/or *h*GGPPS enzymes, with apoptosis in osteoblasts and osteoclasts leading to antiproliferative effects.

In osteoblasts, the ZOL-induced activation of the TRPV1 channel counterbalances the antiproliferative effects associated with the inhibition of intracellular *h*FPPS and/or *h*GGPPS enzymes, thereby shifting the concentration–response relationship of the drug to the right on the log concentration axis. This mechanism is not operative in osteoclasts lacking the TRPV1 channel, and explains the osteoclast selective antiproliferative effect of ZOL on these cells.

## 4. Materials and Methods

### 4.1. Cell Culture

Preosteoblast-like cells from mouse MC3T3-E1 (ECACC 99072810) and preosteoclast-like cells from mouse RAW264.7 (ECACC 91062702) were cultured in Minimum Essential Medium Eagle-Alpha Modification (αMEM) supplemented with 10% fetal bovine serum (FBS), 1% L-glutamine, and 1% antibiotics (penicillin–streptomycin), under standard conditions, at 37 °C, in a humidified atmosphere containing 5% CO_2_. Both cell lines were certified by the European Collection of Authenticated Cell Cultures (ECACC) and provided by Sigma-Aldrich (Milan, Italy). Cells were provided at P4 and used from P5 to P10. Differentiated MC3T3-E1 cells for the mineralization assay were cultured in the mineralization medium (MM) obtained by the addition of 50 µg/mL ascorbic acid and 10 mM β-glycerophosphate (Sigma-Aldrich, St. Louis, MO, USA) [36,37].

### 4.2. Ex-Vivo Culture of Native Mouse and Rat Mesenchymal Stem Cells

Native mouse and rat mesenchymal stem cells were obtained as previously described [33]. Briefly, cells were isolated, under sterile condition, from the tibia and femoral marrow compartments and cultured in Dulbecco’s Modified Eagle’s Medium (DMEM) supplemented with 10% fetal bovine serum (FBS), 1% L-glutamine, and 1% antibiotics (penicillin–streptomycin) under standard conditions, at 37 °C, in a humidified atmosphere containing 5% CO_2_. Non-adherent cells were carefully removed after 3 h of incubation time. After primary cultures had become almost confluent, they were treated using Trypsin-EDTA Solution 1× (0.25% trypsin, 0.02% EDTA) for 2 min at room temperature. The same protocol was applied 3 times, so that a purified population of MSCs was obtained 3 weeks after the initiation of culture, and then used for experiments. Mouse and rat bone marrow-derived osteoblasts for the mineralization assay were cultured in the mineralization medium.

### 4.3. Ethical Statements

In accordance to 3R RULE (DIRECTIVE 2010/63/EU) Replace, Reduce, Refine, bone marrow cells were collected both from wild-type male mice C57BL sacrificed for other scientific purposes (Ethic Committee name, Organization for Animal Health O.P.B.A. University of Bari, Italy, project: DGSAF0024012) and from male Wistar rats (Ethic Committee name, Organization for Animal Health O.P.B.A. University of Bari, Italy, project: 7282018PR). Patch clamp experiments on *Xenopus laevis* oocytes were performed at Centro Interdisciplinario de Neurociencia de Valparaíso (CINV) (Chile) in accordance with NIH “Guidelines for Egg and Oocyte Harvesting in *Xenopus laevis*” [38].

### 4.4. CCK-8 Intracellular Dehydrogenase Assay

The activity of the intracellular dehydrogenases was evaluated by using the Cell Counting Kit-8 (CCK- 8) (Sigma-Aldrich), a highly sensitive test that utilizes water-soluble tetrazolium salt [34,39]. WST-8 2-(2-methoxy-4-nitrophenyl)-3-(4-nitrophenyl)-5-(2,4-disulfophenyl)-2H-tetrazolium monosodium salt can be reduced by dehydrogenases in cells, producing an orange colored formazan dye soluble in the culture medium. The cells were counted by using Scepter™ 2.0 (Merck Millipore Corporation, New York, NY, USA) cell counter, seeded in 96-well plates at a density of 8 × 10^3^ cells/well, and pre-incubated for 24 h under standard conditions. Then, cells were treated for 72 h with different concentrations of the drug solution. Finally, the activity of the intracellular dehydrogenases was evaluated by adding CCK-8 solution (10 μL) to each well and measuring the absorbance at λ = 450 nm after 2 h of incubation with CCK-8 solution, using the microplate reader Victor^®^ 3V (PerkinElmer^®^, Waltham, MA, USA). The changes of the cell viability were expressed as percentage (%) changes of intracellular dehydrogenase activity induced by the drug with respect to the control.

### 4.5. Mineralization Assay

This assay was performed to evaluate cells’ capability to mineralize, that is, their ability to create calcium deposits or nodules after the expression of a mature osteoblast phenotype, as previously described [6]. Briefly, the cells (1 × 10^6^ cells/mL/well) were cultured in the mineralization medium using 12- and 24-well plates in the presence of different concentrations of the drug solution for approximately 10–15 days under standard conditions. The medium was refreshed every 3 days. After mineralized nodules have become visible, cells were stained with the alizarin red S staining solution 2% (m/v), able to form a bright red stained calcium complex, and washed 3 times with PBS to avoid non-specific dying. For the quantification, 10% acetic acid solution was added to each well to completely elute the dye, and then the absorbance at λ = 405 nm was measured using the microplate reader Victor^®^ 3V. The changes of mineralization ability were expressed as the percentage (%) change of mineralization induced by drugs with respect to the control or with respect to 10^−3^ M NaF, a well-known chemical compound inducing mineralization [13]. For co-incubation experiments, the capability of channel antagonists to inhibit ZOL-induced mineralization was expressed as percentage (%) changes of mineralization induced with respect to ZOL-induced mineralization. For taking pictures, cells were incubated in 6-well plates containing slide glasses; the staining of randomly selected areas was evaluated by using Olympus CX41 microscope, and thereby photographed at the end of the experiment.

### 4.6. Osteoclastogenesis Assay

The TRAP kit (Sigma-Aldrich, St. Louis, MO, USA) was used to evaluate the ability of drugs to inhibit osteoclast proliferation and activity by detecting the presence of tartrate-resistant acid phosphatase in mature osteoclasts. RAW264.7 cells (8 × 10^4^ cells/well) were incubated in 6-well plates containing glass slides to allow photos to be taken at the end of the experiment in a specific medium of differentiation containing 50 ng/mL RANKL. The medium was refreshed every 2 days. After 7 days of differentiation with RANKL, cells were treated with different concentrations of the drug solutions for 72 h; then, they were fixed with 10% formaldehyde/PBS for 15 min, and the plate was washed using deionized water and then stained for TRAP, according to the manufacturer’s instructions. The staining of randomly selected areas was evaluated at the microscopy and thereby photographed. 

### 4.7. Drugs and Solutions

Whole-cell patch clamp experiments on RAW264.7 cells, MC3T3-E1 cells, and native mesenchymal stem cells (MSCs) from bone marrow of mouse and rat were performed in asymmetrical K^+^ ion concentrations. The pipette solution contained:132 mM K^+^-glutamate, 1 mM ethylene glycol-bis(β-aminoethylether)-*N*,*N*,*N′*,*N*′-tetraaceticacid (EGTA), 10 mM NaCl, 2 mM MgCl_2_, 10 mM HEPES, 1 mM Na_2_ATP, 0.3 mM Na_2_GDP (pH = 7.2). The bath solution contained 142 mM NaCl, 2.8 mM KCl, 1 mM CaCl_2_, 1 mM MgCl_2_, 11 mM glucose, and 10 mM HEPES (pH = 7.4) [40,41]. For experiments on MC3T3-E1 in low Cl^−^, 142 × 10^−3^ M of NaCl was replaced by Na-glutamate. CaCl_2_ was added to the pipette solutions to give a free Ca^2+^ ion concentration of 1.6 × 10^−6^ M in whole-cell experiments. The calculation of the free Ca^2+^ ion concentration in the pipette was performed using the MaxChelator software (Stanford University, Stanford, CA, USA) [42,43].

For macropatch experiments on *Xenopus* oocytes expressing TRPV1 channel, the same solution was used both for the pipette and external recording solution: 150 mM NaCl, 10 mM EGTA, 2 mM MgCl_2_, and 10 mM HEPES (pH = 7.4) [44].

Channels modulators iberiotoxin (Ibtx), tetraethylammonium hydrochloride (TEA), Ba^2+^ ions, ruthenium red (RR), capsaicin (Caps), and capsazepine (Capsz) were all purchased from Sigma (SIGMA Chemical Co., Mi, Italy). The three bisphosphonates (BPs), zoledronic acid (ZOL), alendronate (ALE), and risedronate (RIS), were synthetized and purified in our labs as previously described [6].

Stock solutions of Ibtx, TEA, Ba^2+^, and Capsz were prepared by dissolving the drugs in dimethyl sulfoxide (DMSO), RR was solubilized in water, and Caps was dissolved in ethanol, at concentrations of 118.6 × 10^−3^ M. BPs were provided as a stock solution in phosphate-buffered saline (PBS) at 1 or 10 mM. Stock solutions were stored in the refrigerator, whereas dilute solutions were directly prepared on the day of the experiments and maintained at room temperature. For cell proliferation assays, diluted solutions of drugs were prepared using the medium. For patch clamp experiments, microliter amounts of the stock solutions were added to the bath solutions as needed. DMSO did not exceed 0.07%; at this concentration, this solvent does not normally affect current or cell proliferation. 

### 4.8. Whole-Cell Recordings in the Cells

Drug actions on the channel currents recorded during instantaneous I/V relationships were investigated by applying a depolarization protocol, in a range of potentials going from −100 to +100 mV (Vm) for RAW264.7 cells, and from −80 to +120 mV (Vm) for MC3T3-E1 cells in 10 mV steps, starting from HP = −60 mV (Vm), in physiological conditions, with asymmetrical K^+^ ion concentrations (int K^+^: 132 × 10^−3^ M; ext K^+^: 2.8 × 10^−3^ M) using whole-cell patch clamp technique. For mesenchymal stem cells from mouse and rat bone marrow cells, the current was obtained in response to voltage pulses from −90 to +70 mV (Vm) in 20 mV steps. Currents are expressed as densities (pA/pF) to control for cell size/capacitance differences. All experiments were performed at room temperature (20–22 °C) and sampled at 2 kHz (filter = 1 kHz) using an Axopatch-1D amplifier equipped with a CV-4 head-stage (Axon Instruments, Foster City, CA, USA). Patch pipettes were pulled from PG52165-4 Patch Clamp #8250 Glass Capillaries, 1.65mm o.d., 1.1 mm i.d. (World Precision Instruments) with a vertical puller (PP-82 Narishige Tokyo, Japan) to give a resistance of 8–10 MΩ. Data acquisition and analysis was performed using pCLAMP 10 software suite (Axon Instruments, Foster City, CA, USA) as previously described [45,46,47]. Seal resistance was continuous monitoring during the experiment; cells not showing a stable seal (R >> 1 GΩ) were not selected for further analysis. 

### 4.9. Heterologous Protein Expression Electrophysiology

For patch clamp experiments in *Xenopus* oocytes, the transcribed cRNA for TRPV1 channel was injected into oocytes using a Nanoliter 2010 WPI (World Precision Instruments, Sarasota, FL, USA) microinjector. Then, they were incubated in ND96 (96 mM NaCl, 2 mM KCl, 1 mM CaCl_2_, 1 mM MgCl_2_, 5 mM HEPES, pH 7.6) solution at 18 °C for 2–3 d after injection [48]. Patch pipettes were pulled in a horizontal pipette puller (Sutter Instruments) from borosilicate capillary glass (World Precision Instruments; 1B150F-4), and pipette tips were fire-polished using a heating filament under a microscope to promote high-resistance seal formation [48,49].

Inside-out excised patch recordings were performed at 20° using patch pipettes with a tip size of 15–25 µm (pipette resistance <1 MΩ after polishing). Currents were obtained in response to voltage pulses from −60 to +260 mV (Vm) in 10 mV steps and were recorded with an *Axopatch 200B* system (Axon Instruments) using Clampex 10 (Axon Instruments) acquisition software. Leak subtraction was performed based on a P/4 protocol [50]. Electrophysiological data were analyzed with Clampfit 10.5 software (Molecular devices corporation, Sunnyvale, CA, USA).

### 4.10. Data Analysis and Statistics

Data were collected and analyzed using Excel software (Microsoft Office 2010); curve fitting was made using SigmaPlot 10.0. The statistical results are presented as mean ± SEM. The number of replicates relative to each experimental dataset was reported in the results paragraph and in the figure description. The Student *t*-test was used to evaluate the significance of differences between the means of two groups. *p* values < 0.05 were considered to indicate statistical significance.

For patch clamp experiments on cells, the percentage of activation was calculated as (I drug − I CTRL)/(I max − I CTRL), where I max was evaluated at the maximum voltage applied in the control (CTRL) condition. For patch clamp experiments on *Xenopus* oocytes, the percentage of activation of ZOL was calculated as (I drug − I CTRL)/(I Caps − I CTRL), referring to the amount of currents activated by the TRPV1-agonist capsaicin (10^−5^ M) at the same voltages.

## 5. Conclusions

TRPV1 channel can be a novel target for zoledronic acid and structurally related BP compounds, mediating the proliferative cytoprotective action of these drugs on osteoblasts. Further investigations are needed to clarify the binding site/s of this class of drugs on the TRPV1 channel. This mechanism may contribute to the anti-osteoporotic effect of zoledronic acid, as well as the pain-relieving effect. Beneficial effects of zoledronic acid can be expected in skeletal muscle where TRPV1 channel appear to play a role in activating hypertrophic signaling and cytoprotection [51,52].

## 6. Patents

Currently no patents are resulting from the work reported in this manuscript.

## Figures and Tables

**Table 1:** Fitting parameters of the concentration–response relationships of percentage reduction of dehydrogenase activity vs. BP concentration in preosteoclast RAW264.7 and preosteoblast MC3T3-E1. Values are expressed as the mean ± SEM of at least three replicates, as evaluated by using SigmaPlot 10. Data significantly different vs ZOL data *.

Drugs	RAW264.7 E_max_ (%)	RAW264.7 IC_50_ (M)	RAW264.7 Hill Slope	MC3T3-E1 E_max_ (%)	MC3T3-E1 IC_50_ (M)	MC3T3-E1 Hill Slope
ZOL	−77.07 ± 5.63	2.62 × 10^−7^ ± 3.21 × 10^−8^	0.77 ± 0.1	−78.88 ± 7.54	2.02 × 10^−5^ ± 7.70 × 10^−6^	1.44 ± 0.19
ALE	−59.77 ± 5.64 *	5.87 × 10^−8^ ± 1.64 × 10^−9^	0.82 ± 0.1	−77.28 ± 3.49	9.98 × 10^−6^ ± 1.07 × 10^−7^	1.67 ± 0.11
RIS	−69.25 ± 9.57	5.35 × 10^−7^ ± 1.08 × 10^−8^	0.41 ± 0.08	−75.33 ± 5	1.97 × 10^−5^ ± 5.45 × 10^−7^	1.75 ± 0.2
